# Transcriptome profiling for developmental stages *Protaetia brevitarsis seulensis* with focus on wing development and metamorphosis

**DOI:** 10.1371/journal.pone.0277815

**Published:** 2023-03-01

**Authors:** Jihye Hwang, Eun Hwa Choi, Bia Park, Gyeongmin Kim, Chorong Shin, Joon Ha Lee, Jae Sam Hwang, Ui Wook Hwang

**Affiliations:** 1 Department of Biology Education, Teachers College and Institute for Phylogenomics and Evolution, Kyungpook National University, Daegu, Korea; 2 Phylomics Inc., Daegu, South Korea; 3 School of Life Sciences, Graduate School, Kyungpook National University, Daegu, South Korea; 4 School of Industrial Technology Advances, Kyungpook National University, Daegu, South Korea; 5 Department of Agricultural Biology, National Institute of Agricultural Sciences, Rural Development Administration, Wanju, South Korea; 6 Institute for Korean Herb-Bio Convergence Promotion, Kyungpook National University, Daegu, South Korea; Central University of Punjab, INDIA

## Abstract

A white-spotted flower chafer *Protaetia brevitarsis seulensis* widely distributed in Asian countries is traditionally used in oriental medicine. This study explored gene expression abundance with respect to wing development and metamorphosis in *P. b. seulensis* based on the large-scale RNA-seq data. The transcriptome assembly consists of 23,551 high-quality transcripts which are approximately 96.7% covered. We found 265 wing development genes, 19 metamorphosis genes, and 1,314 candidates. Of the 1,598 genes, 1,594 are included exclusively in cluster 4 with similar gene co-expression patterns. The network centrality analyses showed that wing development- and metamorphosis-related genes have a high degree of betweenness centrality and are expressed most highly in eggs, moderately in pupa and adults, and lowest in larva. This study provides some meaningful clues for elucidating the genetic modulation mechanism of wing development and metamorphosis in *P. b. seulensis*.

## Introduction

*Protaetia brevitarsis seulensis* (Coleoptera: Scarabaeiformia: Scarabaeidae: Cetoniinae), commonly known as the white-spotted flower chafer, is widely distributed in Korea and southeast Asia, including China [1, 2]. It belongs to the major insect family Scarabaeidae, which are distributed globally with a high level of diversity comprising nearly 6,200 species and almost 267 genera [3]. Its larva live on the ground and feed on rotten food. This type of habitat and diet exposes them to numerous types of pathogenic microbes imposing strong selective pressures on their immune systems [3, 4]. In Korean traditional medicine, its larva are used to treat microbial infections [5]. Moreover, this insect contributes the highest revenue among edible insects.

The draft genome of *P. b. seulensis* habituated in China and South Korea has been reported in previous studies [6, 7]. In China, Illumina and PacBio sequencing platforms have been used to generate a genome total of 135.75 gigabases (Gb), providing 150-fold coverage based on the 810-megabases (Mb) estimated genome size. The assembled *P. b. seulensis* genome was 751 Mb (including the scaffolds longer than 2 kilobases (kb)) with 327 scaffolds, and the N50 length of the assembly was 2.94 Mb. Further, 34,110 genes (22,229 in scaffolds and 11,881 located in alleles) were identified using Evidence Modeler. In South Korea, we sequenced *P. b. seulensis* genome using Illumina NextSeq500 and PacBio sequencing platforms, generating 277.7 gigabases (Gb), providing 190-fold coverage based on the 435-megabases (Mb) estimated genome size. The assembled *P. b. seulensis* genome was 692 Mb (including the scaffolds longer than 2 kilobases (kb)) with 302 scaffolds, and the N50 length of the assembly was 4.53Mb. A total of 19,371 genes were identified using a gene prediction algorithm.

The transcriptome represents all genes expressed in a cell or a population of cells, providing a biological perspective on cellular processes. Novel high throughput sequencing technologies greatly facilitate the global analysis of the blueprint of development-related genes [8, 9]. Transcriptome analysis based on RNA-seq can effectively identify the temporal and spatial unique gene expression patterns in organisms [10]. RNA-seq has been widely used to reveal biological phenomena, gene expression profiles, and gene discovery of insects [11–13]. Quantified transcriptome analysis can effectively help us understand insect feeding mechanisms [13], defense [14], development process [15], and host-pathogen interaction of insects [16]. However, in insects, comparative transcriptome analysis related to its development has been only applied to several species, including *Glyphodes pyloalis* [17], *Cryptolaemus montrouzieri* [18], and *Bombyx mori* [19]. The comparative transcriptomics studies between *D. melanogaster* and *Blattella germanica* reported general trends in modern insect evolution [20]. Therefore, this is the first transcriptome study of the developmental stages of eggs, larva, pupae, and adults of *P. b. seulensis*.

Identification of specific genes regulating wing development would provide insights for further study of the molecular mechanisms controlling metamorphosis development. Wing development and metamorphosis signaling pathways are highly evolutionarily conserved in different animals [21, 22]. However, current knowledge about insect wing development mechanisms is primarily from the *D. melanogaster* [24–26]. Knowledge about the identification and involvement of wing development-related genes in various insects, especially in *P. b. seulensis*, remains unclear and incomplete. By comparing the *P. b. seulensis* transcriptome according to the four different developmental stages (eggs, larva, pupa, and adults), we determined the specific genes and related candidates involved in the wing development and metamorphosis.

This study enhanced the understanding of wing development and metamorphosis in *P. b. seulensis* during its developmental period. Differentially expressed genes (DEGs) were identified. Their functions were analyzed based on the various types of databases. Protein-protein interaction network was constructed by applying the concepts of interlog to explore gene importance and their potential functions based on the network topology. Furthermore, these investigations were focused on developmental regulatory factors responsible for wing development and metamorphosis in *P. b. seulensis* focusing on the network system. These data can increase the understanding of the development of *P. b. seulensis*. By performing comparative analyses of the gene expression levels and different developmental stages of *P. b. seulensis*, we aimed to determine and discuss novel gene sets related to the wing development and metamorphosis in *P. b. seulensis*, possibly revealing insights at the molecular level.

## Materials and methods

### Insect sample collection

The white-spotted flower chafer (*P. b. seulensis*) managed by the insect breeding facility of the National Academy of Agricultural Sciences (Wanju, Republic of Korea) was used in this study. Larva and adults were raised on fermented oak sawdust in a dark photoperiod continuous breeding room at 28℃±1℃, less than 70% relative humidity (RH). For samplings, three egg lumps, each consisting of 20 eggs, three third instar larva, three 15-day-old pupae, and three 10-day-old adults were randomly selected.

### RNA preparation for sequencing

Total RNA at four different stages of development (eggs, larva, pupa, and adults) was isolated for overall transcriptome sequencing. During the total RNA isolation, the larva were washed with PBS, sterilized with 70% ethanol, and anesthetized on ice. The whole body was fixed, the dissected skin was cut along the abdomen, and tissue was collected. From the prepared samples, we extracted mRNAs with TRIzol reagent (Invitrogen, Carlsbad, CA, USA). RNA quantification and quality check were carried out using ultraviolet absorption and gel electrophoresis. All the samples were washed with 70% ethanol to reduce microbial contamination from the surface. Fine powders in liquid nitrogen, excluding eggs, were quickly crushed after ethanol volatilization. During the extraction of eggs, three pestles were homogenized in a 1.5 ml tube containing TRIzol reagent. RNA quantification was carried out using ultraviolet absorption and gel electrophoresis was used to confirm its quality.

### cDNA library preparation

The mRNA sequencing library was prepared according to the Illumina TruSeqRNA Sample Preparation Kit v2 (Illumina, Inc., San Diego, USA). Total RNA of 1ug was used in poly mRNA selection using streptavidin-coated magnetic beads, followed by thermal decomposition of the selected mRNA. Subsequently, 200–500 bp fragmented mRNA was used as a template for cDNA synthesis using reverse transcriptase with random primers. The cDNA was converted into finally repaired double-stranded DNA and incorporated into 15 cycles of specific index adapters for multiplexing, purification, and amplification. The quality and functionality of the final amplification library were tested by Agilent 2100 BioAnalyzer (Palo Alto, USA) that uses highly sensitive DNA chips. The library was prepared following the Illumina Protocol. Random fragmentation of DNA or cDNA and binding of 5’ and 3’ adapters were also investigated. Alternatively, fragmentation and nodule responses could be integrated into one single stage, considerably improving the efficiency of library preparation. The adapter binding fragments were then PCR-amplified and gel purified. Further, the concentrations of each library were measured by real-time PCR. Agilent 2100 Bioanalyzer was used to profile the distribution of insertion sizes. To generate clusters, the library was loaded into a flow cell and fragments were captured on a surface-coupled oligo lawn that complements the library adapter. Subsequently, the fragments were amplified to different clone clusters by bridge amplification. After cluster generation, Illumina HiSeq was used to sequence the cluster library. Based on the manufacturer’s instructions (Illumina Inc., USA) and pair-end sequencing, the TruSeq Rapid SBS kit or TruSeq SBS kit v4 was used for 100 cycles. The sequencing protocol used was HiSeq.TM2500 System User Guide #15011190 Revised VHCS 2.2.58.

### RNA-seq analysis

HiSeq was used to generate a sequence of raw images. TM control software (HCS v.2.2.) and basic calls were made through integrated primary analysis software called RTA (v.1.18). The HiSeq-generated image TM2500 was converted into a nucleotide sequence by base call and stored in FASTQ format using the Illumina package bcl2fastq (v.1.8.4). Clean reads were generated by filtering dirty reads, including adapters, unknowns, or low-quality score bases, from raw ones. Clean leads were Remapped to the *P. b. seulensis* genome from China and gene sequences using the existing tuxedo protocol [28]. Mismatch in reading below the fifth base was allowed in alignment. Readings that match the reference rRNA sequences were also mapped and deleted. We used Tophat2 software and cufflinks packages (v.2.2.1) to calculate the FPKM (fragments per kb per million reads) values for each gene used in subsequent data analysis.

### Genome size estimation and assembly

The isolated RNA was sequenced using ISO-Seq sequencing methods familiar with long and short-read sequences. DNALink, a Korean service provider, carried out a complete experimental process. The Illumina pair-end sequence initially received technical artifacts (i.e., basic call errors (PHERD quality scores (Q)) and adapter filtering using the Trimmomatic-0.32 method [26]. Finally, jellyfish v2.0 was used to estimate the size of the genome, whereas its depth and size were calculated and explained in the Thai genome article [27]. These Illumina readings were also used to correct errors in PacBio readings by clc-assembly-cell v5.1.184548–20181101136. Finally, the modified PacBio readings were used in the initial draft version of the Kolbe genome using Falcon-Unzipv0.30 and haplotype assembler [28]. The constructed contigs were evaluated for completeness using BUSCO v.3.0 with the insect (odb9) reference dataset [29].

### Gene prediction and annotation

The genes in the draft Kolbe were predicted using the in-house gene prediction pipeline, including the evidence-based gene modeler (EVM; includes Exonerate [30], AUGUSTUS [31], and GENEID [32]), ab-initio gene modeler, and consensus gene modeler. Two transcripts (i.e., Illumina (132.8Gb) and Isosek (0.7Gb)) were mapped to the Kolbe repeatedly masked genome, respectively, Tophat, the Cufflink [33, 34] and PASA [35] represented the transcript and gene structure boundaries. The final gene and transcription model were optimized with EVidenceModeler [36] and the agreed-upon gene modeler. Functional annotations (i.e., gene ontology (GO), KEGG pathway) of the final models were obtained using the Blast2GO method [37].

### Gene expression pattern analysis

Differential Gene Expression and Trend Analysis was conducted. Differential Unigene abundances were determined by performing independent alignments of short reads from four libraries against the set of *P. b. seulensis* unigenes using Blast software. Reads per kb per million reads (RPKM) were calculated as RPKM = (1000000*C)/(N*L/1000), where C = number of mappable reads in specific unigenes, N = total number of reads mapped to unigenes in a particular sample, and L = length of the unigene19 (see S1 Table). During the analyses of egg expression profiling, and the larva, pupa, and adults based on RPKM values, Short Time-series Expression Miner (STEM) software (http://www.cs.cmu.edu/jernst/stem) was used to compare the trends exhibited in these four stages [38]. P-values corresponded to the differential gene expression test, which was performed to analyze all trends in these four stages.

### Construction *P. b. seulensis* protein interaction network

The *P. b. seulensis* protein interaction network was constructed by mapping the interaction information (Flybase) of *D. melanogaster* into the *P. b. seulensis* species. The betweenness centrality of the node in the *P. b. seulensis* network with high centrality nodes signifies the important nodes in the signaling flow of the entire network.

### Comparative and phylogenetic analyses

To analyze gene family evolution in coleoptera, all-to-all BLASTP analysis was performed followed by Markov clustering to identify orthologous gene groups with OrthoMCL v.2.0.9 (https://github.com/apetkau/orthomclsoftware-custom), according to the standard protocol using a default inflation number of 1.5. We constructed a phylogenetic tree using IQ-Tree 1.5.1 (http://www.iqtree.org/release/v1.5.1/). A maximum likelihood phylogenetic tree was inferred from a concatenated amino acid data set containing 9,608 one-to-two orthologous genes shared by all 12 species by IQ-Tree 1.5.1 with the best fit model (LG+F+G4). In total, 269 wing development-related genes and 19 genes for metamorphosis were identified. We used conserved genes in five species within 12 coleoptera species. the half gap option of Gblock.

## Results

### *De novo* sequencing and assembly of the *P. b. seulensis* transcriptome

To determine molecular mechanisms for developmental changes along the *P. b. seulensis* life stages, sequenced cDNA libraries representing the four different developmental stages were constructed from the eggs (accession number: SAMN15647968), larva (SAMN15647969), pupa (SAMN15647970), and adults (SAMN15647971) using the Illumina HiSeqTM 2000 sequencing platform. A total of -692.7 Mb reads (Table 1) were generated and finally assembled into 23,551 unigenes with an average length of -3 Mb and the N50 length of -5Mb. The cDNA libraries produced a total of ca. 193.5 Mb clean reads, representing most data with the Q20 score of over 95%. Quality checks and Unigene assembly were carried out using Trinity version 1.0. software [39].

**Table 1:** Result of the genome assembly of *P. b. seulensis*.

Category	Size
NCBI taxonomy ID	438893
Estimatedgenomesize (bp)	656,797,776
No. Contigs	224
Draft genome size (bp)	692,712,625
Coverage (%)	105.5
Avg. length (bp)	3,092,467
Min. length (bp)	26,261
Max. length (bp)	16,895,244
N50	4,997,170
N (%)	0.00
GC (%)	33.42

### Annotation of predicted proteins from the *P. b. seulensis* transcriptome

The unigenes obtained for *P. b. seulensis* were annotated as hypothetical proteins using BLASTX tools based on various publicly released protein databases such as UniProt and Ensembl [40–42]. Out of 23,551 unigenes, 15,667 (ca. 66.52%) were specifically matched and annotated based on Blast hits (Table 2). The remaining 7,884 unigenes (ca. 33.48%) were matched in the non-redundant database. Additionally, the 10,844 unigenes (ca. 46.04%) had significant matches in the Gene Ontology (GO) database. Additionally, the 8,565 (ca. 36.37%) and 8,474 (ca. 35.98%) unigenes had specific matches in the COG and KEGG databases, respectively.

**Table 2:** Annotation of genes of the *P. b. seulensis*.

Category	Number of genes	Coverage(%)
Total	23,551	100
No hits	7,884	33.48
blast hits	15,667	66.52
GO	10,844	46,04
KEGG	8,474	35.98
COG	8,565	36.37
pFAM	10,821	45.95
SignalP	1,553	6.59
TmHMM	3,227	13.70

### RNA-seq and gene expression profiling of the *P. b. seulensis* transcriptome

**Fig 1:**
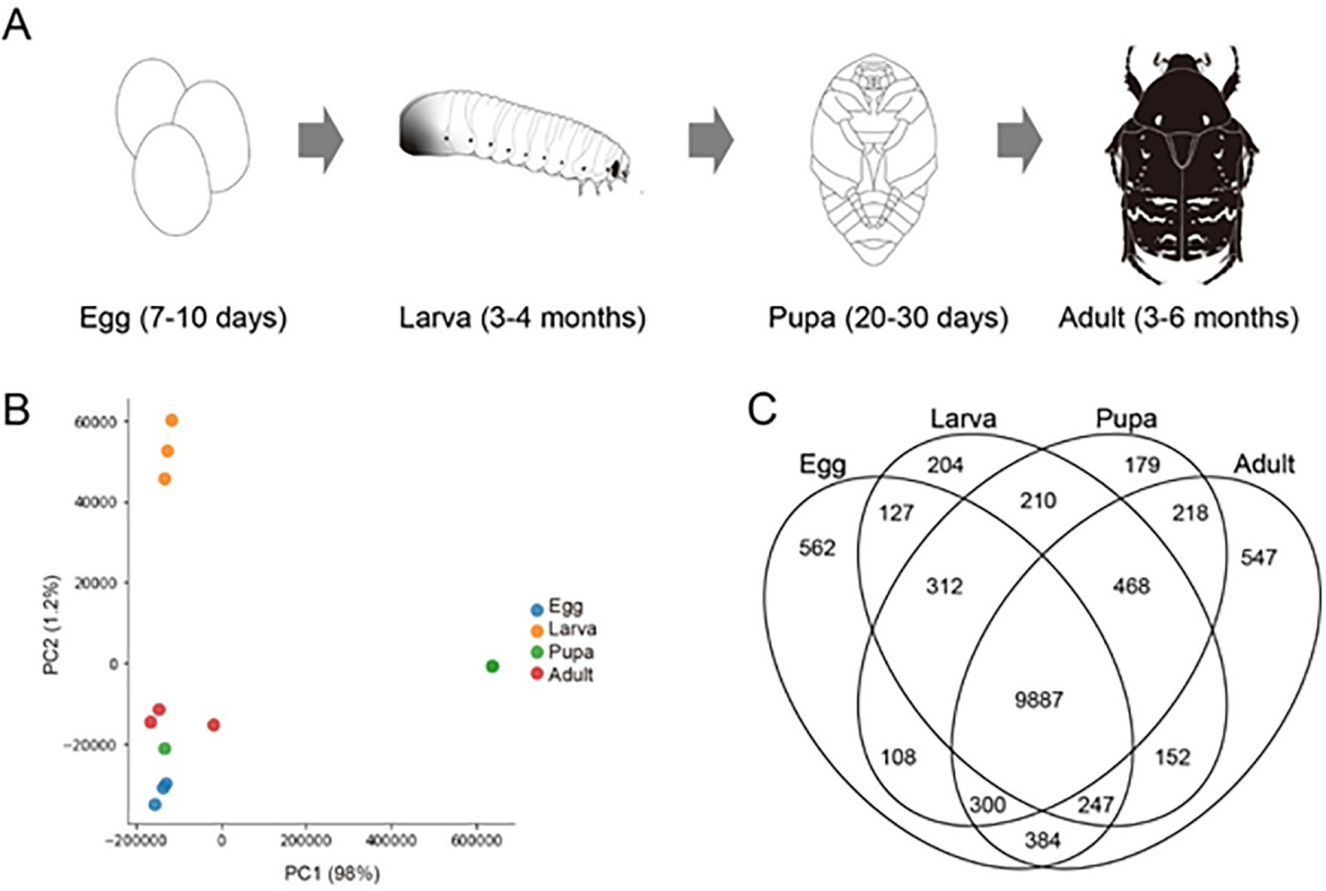
Quality assessment of *P. b. seulensis* RNA-sequencing (RNA-seq) data. (**a**) 4 development stages of *P. b. seulensis* for RNA-seq. (**b**) Grouping of samples via principal component analysis based on the *P. b. seulensis* transcriptome expression abundance. (**c**) Venn diagram shows the unique and shared unigenes during *P. b. seulensis* development.

To identify wing development and metamorphosis-related genes, RNA-seq analysis of the samples of *P. b. seulensis* along the four developmental stages, including 15–20 eggs, three larva, three pupae, and three adults, was performed (Fig 1A). The quantitative value of each transcript abundance was used to perform a principal component analysis (PCA) (Fig 1B). It was therefore calculated in every stage of the eggs, larva, pupa, and adults, respectively, and the results were depicted on a PCA plot, based on the fundamental differences in the calculated RNA abundance among each other. The results divided the four stages into three different groups on the plot connoting those of the larva (yellow dots) and pupae (a green dot) distinctly separated from the major group, including those from eggs, adults, and a part of the pupae. Next, to explore the tendency of RNA expression, we examined the genes that are commonly expressed in all four developmental stages of *P. b. seulensis*, differentially expressed along the four stages. The result of the intersection analysis (Fig 1C) indicated that 9,887 genes were commonly expressed in all four stages. Furthermore, the degree of expression abundance of stage-specific transcripts appeared as eggs (562), adults (547), larva(204), and pupa (179) sequentially. The three stages except the eggs shared 468 transcripts; the three stages except for pupae 247 transcripts; the three except for larva 300; and with the exception of the adult stage, 312 transcripts. The largest one shared between the two stages was ironically in the pair of eggs and adults (384).

### Identification of the differentially expressed genes (DEGs)

Subsequently, through a two-way analysis of variance (ANOVA), the differential expression along the stages was obtained, as listed in Table 3. To compare the differential expression levels of genes along the stages, up- and down-regulated gene numbers were calculated between every pair of the gene and between every pair of the developmental stages of eggs-larva, larva-pupa, pupa-adults, and eggs-adults in *P. b. seulensis* (Table 3, more than 1.2-fold change in expression compared between two developmental stages, ANOVA p-value less than 0.05). A total of 8,701 different genes showed significant expression profile changes during the egg-to-larva transition process, of which 2,189 genes were up-regulated and 6,512 down-regulated in the egg library. In the larva-to-pupa transition process, comparing their transcriptomes revealed that 8,631 were significant DEGs, among which 6,899 were up-regulated and 1,732 down-regulated in the larva library. In the pupa-to-adult process, out of 8,683 DEGs, 5,798 were up-regulated and 2,885 down-regulated in the pupae library. In the egg-to-adult, 8,692 DEGs were observed, consisting of 5,803 up-regulated and 2,889 down-regulated genes in the adult transcriptome. Furthermore, 2,189 and 6,152 genes were up-and down-regulated, respectively, at the larva stage compared with the egg stage (Eggs versus larva). In addition, 5,838 and 2,861 genes were significantly up-and down-regulated, respectively, at the pupa stage, compared with the egg stage (eggs versus pupa). Further, 1,954 and 6,340 genes were up-and down-regulated, respectively, at the adult stage compared with the larva stage (larva versus adults).

**Table 3:** Number of differentially expressed genes in *P. b. seulensis*.

Stage	Up-regulation	Down-regulation
Egg vs. Larva	2,189	6,152
Egg vs. Pupa	5,838	2,861
Egg vs. Adult	5,803	2,889
Larva vs. Pupa	6,899	1,732
Larva vs. Adult	1,954	6,340
Pupa vs. Adult	5,798	2,885

### Trend analysis

**Fig 2:**
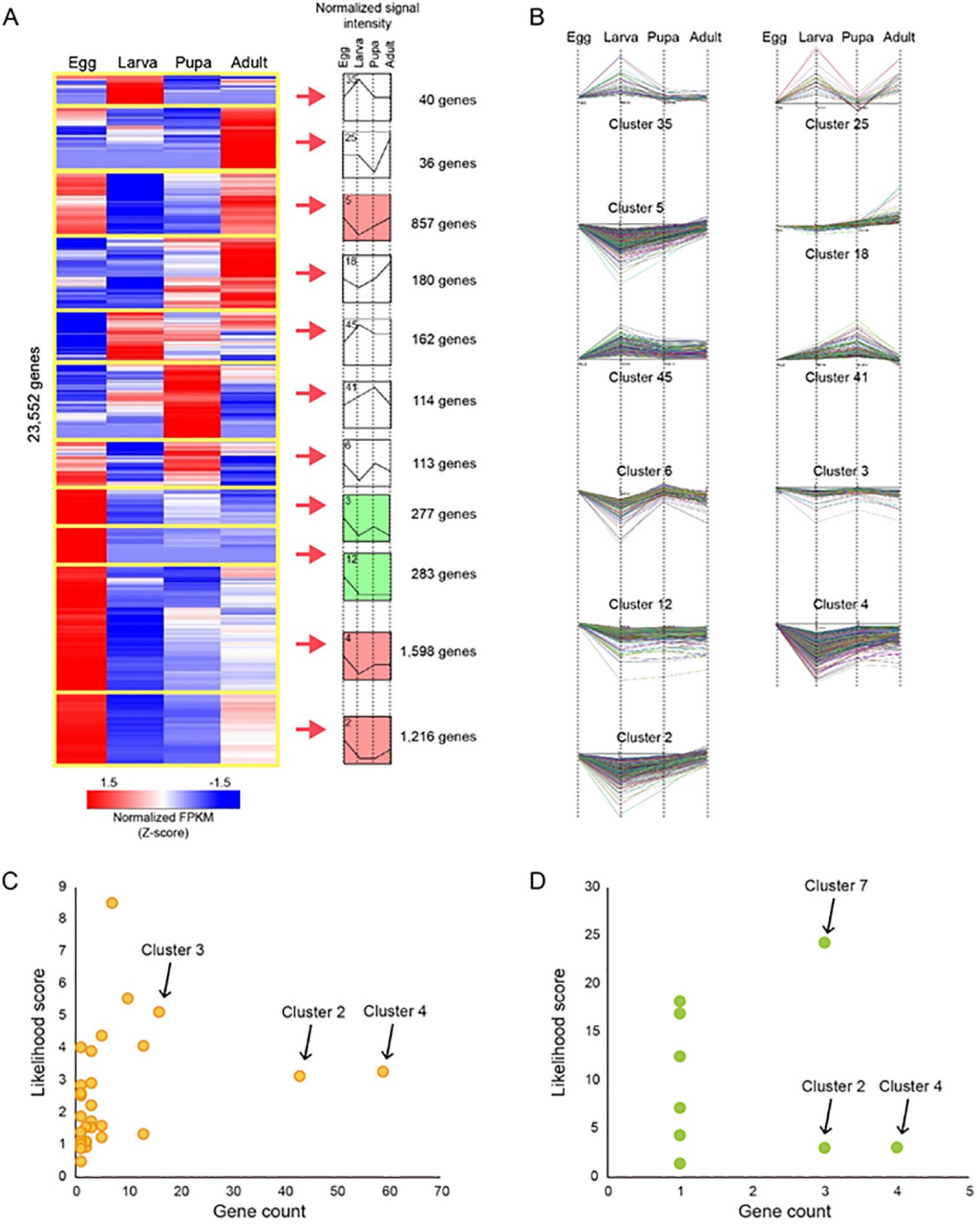
Trend analysis based on gene expression abundance. (**a**) Heatmap shows 23,552 transcripts. The gene counts were normalized into Fragments Per Kilobase of transcript per Million mapped reads (FPKM), then averaged by life stage. The FPKM values for each gene were transformed and then hierarchically clustered using Pearson correlation. Clustering was performed based on similarities between gene expression profiles throughout the developmental stages. The mean expression values for each gene at each of the four developmental stages were normalized to the mean expression values in the former developmental stages to represent expression levels. Groups were chosen based on similar expression patterns over 0.7 of the Pearson correlation coefficient. After clustering the expression profiles, the number in the top left-hand corner of a profile box is the profile ID number. We assigned the number of genes on the right side of each cluster. Nonwhite profiles of the same color represent profiles grouped into a single cluster. White profiles represent the statically non-significant profiles of gene expression. (**b**) Detailed expression profiles of the genes in each cluster. Selected profiles were statically significant. (**c**) Likelihood score and gene count analysis of each cluster’s wing development-related genes. The likelihood score is calculated based on the observation values divided by the expectation values. (**d**) Likelihood score and gene count analysis of the metamorphosis-related genes in each cluster.

Using Short Time-Series Expression Miner software (STEM; [38]) for the gene group clustering based on the expression pattern similarity, it was found that the 9,887 DEGs inferred from the expression data of the four different developmental stages (e.g., eggs, larva, pupa, and adults) were clustered into 49 profiles (Fig 2). Furthermore, 4,876 clustered into 11 profiles (p-value less than 0.05) exhibited down-regulated patterns at Clusters 2, 3, 4, 5, 6, 12, and 18 (Fig 2). In Clusters 2, 3, 4, 5, and 6, 4,028 genes showed down-regulated patterns during the transition from eggs to larva but up-regulated during the larva-to-pupa transition. Interestingly, the 283 DEGs, shown in Cluster 12, were down-regulated only for the hatching stage from eggs to larvae, but their regulation patterns remained unchanged in the other stages. A time-series correlation of changes in expression identified 666 genes with differential expression patterns in the four developmental stages. According to the mRNA abundance ratios (egg versus larva and larva and pupa), the genes were classified into six groups: three up-up-regulated (Group 1), 422 up-down-regulated (Group 2), two constant-up-regulated (Group 3), 51 constant-down-regulated (Group 4), 25 down-up-regulated (Group 5), and 163 down-down-regulated genes (Group 6) (Fig 2B and 2C). Notably, the expression of 422 transcripts in Group 2 steadily increased during the developmental stages but decreased to the baseline during the four developmental stages (Fig 2B and 2C). In contrast, expression of the 163 transcripts in Group 6 decreased during the four developmental stages and then remained unchanged at the pupa stage (Fig 2B and 2C).

#### 0.1 Metamorphosis and wing development related genes are gathered in cluster 4

Using homologous information of the *D. melanogaster*, the function of the *D. melanogaster* was transferred to assign genes related to wing development and metamorphosis using keyword mining (S2 Table). As a result, 267 wing development-related genes and 19 metamorphosis-related genes were identified (S3 and S4 Tables). The cluster was searched for the largest genes related to wing development and metamorphosis. Cluster 4 contains numerous genes related to wing development and metamorphosis. Furthermore, the extent to which the probability was higher than expected was considered. Finally, Cluster 4 was selected by considering both gene count and expectation values described as a likelihood score at the same time (Fig 2C and 2D).

#### 0.2 Expression profile analysis of the wing-development and metamorphosis-related genes

**Fig 3:**
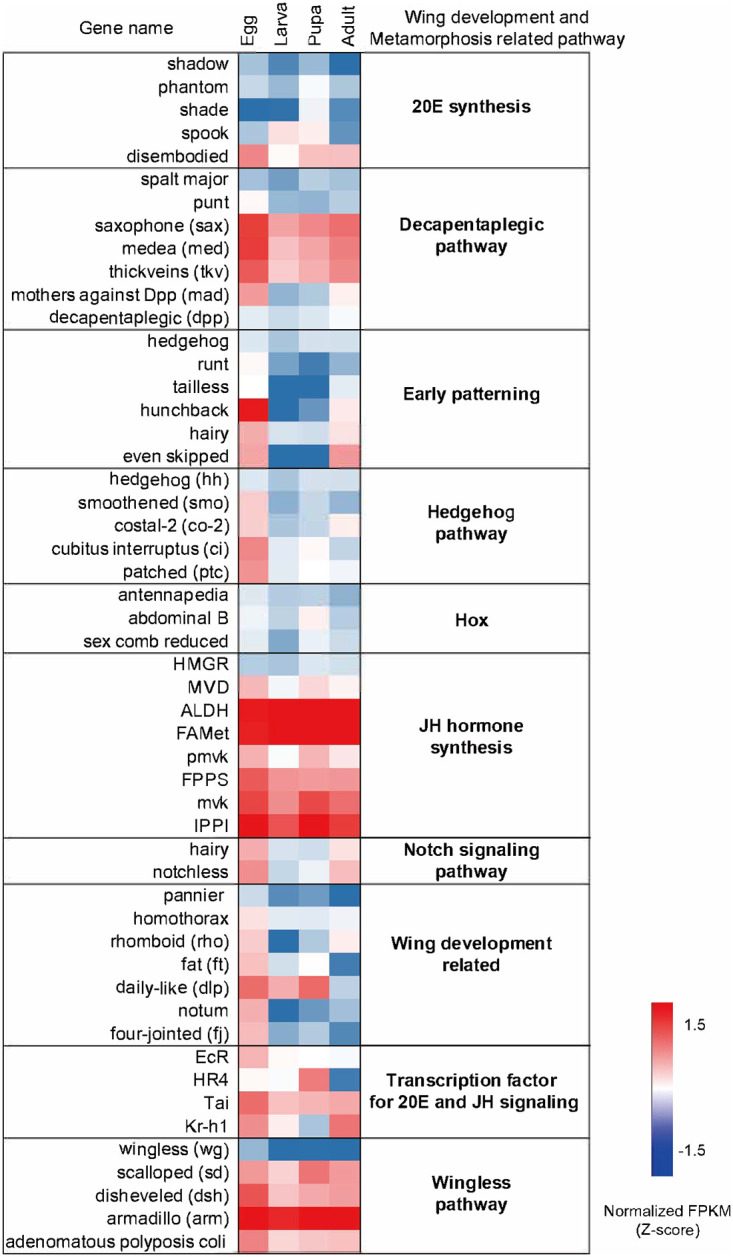
Comparison of *P. b.seulensis* transcriptome profiles of the genes related to development, early development, hox, and metamorphosis. The heatmap of gene expression profiles of 10 major wing development and metamorphosis functional categories. Each column stands for a developmental stage, for example, Egg, Larva, Pupa, and Adult, respectively, and each row represents a transcript. Red stands for high expression abundance, whereas blue represents low expression abundance.

Based on analyses of the pathways, annotation, and reported literature, 57 insect wing development and metamorphosis-related genes were identified [23–25, 43–47]. Considering the development of wing and metamorphosis, several genes were well studied regarding their genetic regulation such as E93, Met, and Kr-h1, which are the components of the MEKRE93 pathway [46]. The gene associated with already known wing formation and metamorphosis shows a high rate of expression in the egg and then reduces the amount of expression as it transitions to the larva. The analysis revealed that the wing development and metamorphosis-related genes showed a similar expression profile to Cluster 4 (Fig 3). In particular, 89% of genes involved in the decapentaplegic pathway, early patterning, hedgehog pathway, wing development-related pathway, and wingless pathway are grouped into Cluster 4. However, scalloped (sd), dishevelled (dsh), armadillo (arm), and adenomatous polyposis coli genes in wingless pathway and saxophone (sax), medea (med), and thickveins (tkv) genes in the decapentaplegic pathway and ALDH, FAMel, pmvk, FPPS, mvk, and IPPI genes in the JH Hormone synthesis pathway were excluded from Cluster 4. These genes are homogeneously overexpressed throughout the developmental stages.

#### 0.3 Function mapping and enrichment analysis of the Cluster 4 genes

**Fig 4:**
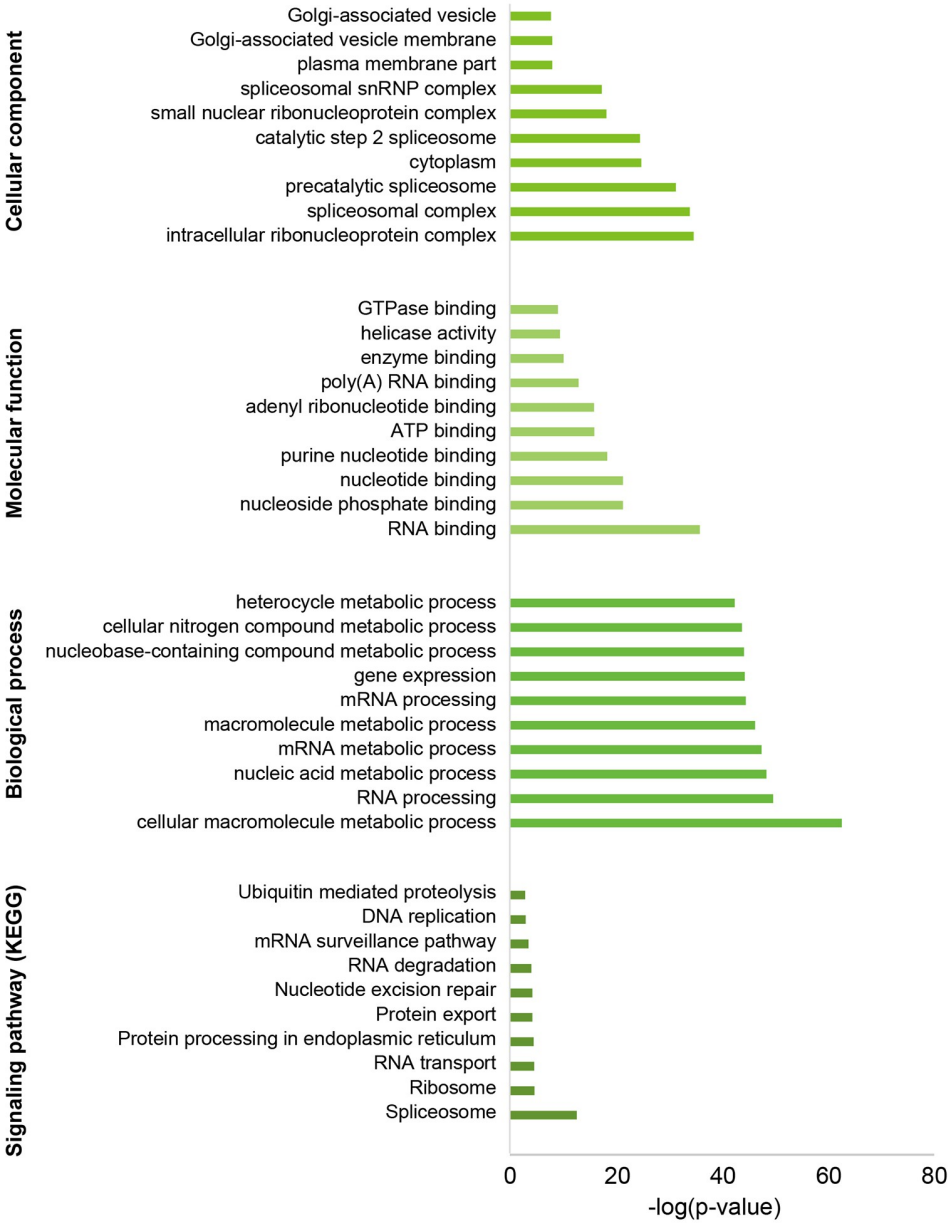
Histogram presentation of the Gene Ontology and KEGG enrichment of the *P. b.seulensis* genes in cluster 4. A total of 1,598 unigenes were allocated to three specific GO categories. 1,183 Unigene sequences were specifically assigned to 236 KEGG pathways. The top 10 enrichment function lists and their *p*-value.

The functional classification of *P. b. seulensis* unigenes was predicted by performing GO and KEGG analyses. A total of 1,598 unigenes were allocated to three specific GO categories: cellular component, biological process, and molecular function. Approximately 1,232 unigenes were putatively identified as having GO functions, including 873 sequences (50.89%) at the biological process level, 432 sequences (27.42%) at the cellular component levels, and 224 sequences (21.69%) at the molecular function level. *P. b. seulensis* unigene sequences mapped to the reference canonical pathways in the KEGG database were analyzed. Moreover, 1,183 unigene sequences were specifically assigned to the 236 KEGG pathways and 164 unigenes were involved in metabolic pathways. These pathways played dominant roles in the following pathways: Spliceosome, Ribosome, RNA transport, Protein processing in the endoplasmic reticulum, and others (Fig 4).

#### 0.4 Network centrality analysis in the *P. b. seulensis* network

Physical and functional links of altered genes were investigated on the assumption that gene pairs that interact or share similar functions tend to interact within the cellular systems [48]. The *P. b. seulensis* network was constructed by transferring the gene interaction network from *D. melanogaster*. To analyze the changes in the mRNA abundances under *P. b. seulensis* developmental stages, the functional association network was generated using 3,126 genes out of 10,254 orthologous genes, based on information on the physical and functional links of these genes. The physical link represents protein-protein interaction, whereas the functional link represents a relationship between two proteins that share a substrate in a metabolic pathway or that are co-expressed, co-regulated, or involved in the same protein complex. The network was also analyzed based on network topological parameters, which are the number of interacting partners (degree) and betweenness centrality. Betweenness centrality reflects the importance of genes in the network, with its score representing the minimum number of links connecting one protein to another in the network. Thus, a high betweenness centrality score implies that genes undergo a greater number of the shortest paths in the network. Therefore, the protein with the highest betweenness centrality score derives considerable importance from its position in the network. Additionally, the number of interacting partners (degree) is important for concluding network centrality.

#### 0.5 Wing development/metamorphosis-related gene identification

**Fig 5:**
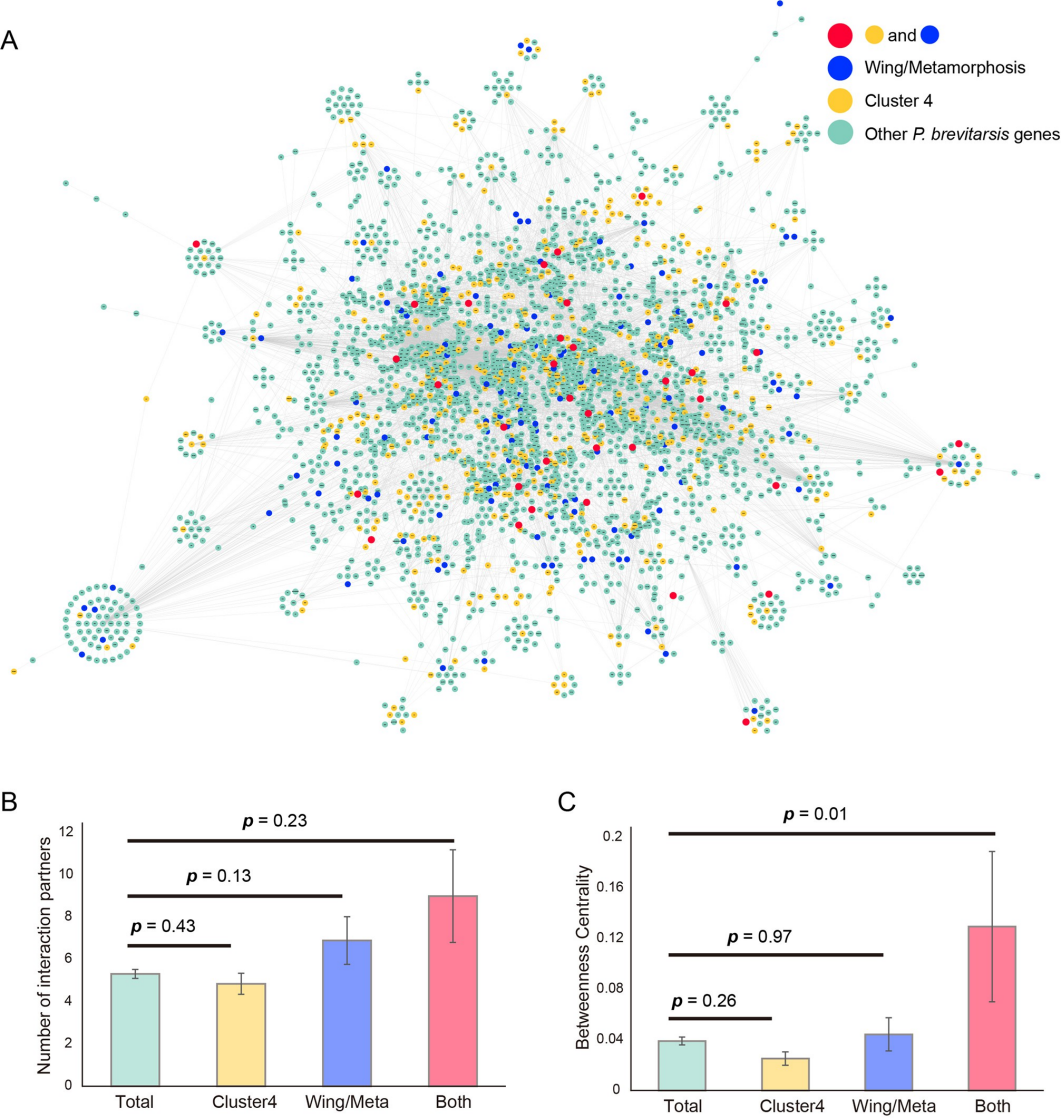
*P. b. seulensis* network. Functional association network analysis. (**a**) Functional association network in *P. b. seulensis*. Nodes in the same group are coded with the same color. Cyan, yellow, blue, and red nodes represent *P. b. seulensis*, cluster 4, wing development and metamorphosis-related, and both cluster 4 and wing development and metamorphosis-related genes, respectively. (**a, c**) The average number of interacting partners and betweenness centrality score of the *P. b. seulensis*, cluster 4, wing development and metamorphosis-related, and both cluster 4 and wing development and metamorphosis-related genes. Statistical analysis was performed using the Mann-Whitney *U* test.

To confirm whether the constructed network contains the gene sets involved in the development of the *P. b. seulensis*, the already known wing development and metamorphosis-related genes were mapped to the *P. b. seulensis* network. Subsequently, 162 Cluster 4 genes were mapped. As a result, 82 wing development and metamorphosis-related genes (30.7%) and 16 both Cluster 4 and wing development and metamorphosis-related genes were mapped in the functional association network (Fig 5A). A comparison of the betweenness centrality score and number of interacting partners among total genes, wing development, and metamorphosis-related genes, and wing development and metamorphosis genes involved in Cluster 4 was conducted. The average betweenness centrality score of known genes involved in Cluster 4 was higher than those of other genes (Mann-Whitney *U* test, *p* value less than 0.05; Fig 4B and 4C). The average number of interacting partners and betweenness centrality of known wing development and metamorphosis genes involved in Cluster 4 was higher than that of other genes. The listed top 10 ranked genes by the betweenness centrality score were selected as possible markers of wing development and metamorphosis (Table 4). Arm (degree, 35, betweenness centrality score, 0.024), Cdc42 (degree 23, betweenness centrality score, 0.012), and RasGAP1 (degree, 18, betweenness centrality score, 0.006) were identified with the highest degree and betweenness centrality scores, possibly serving as molecular bridges to connect other multiple proteins in this network. These genes were previously reported as wing development and metamorphosis-related genes [24]. Genes with high betweenness centrality scores tend to interact with different functional groups and are important for controlling information flow in the network. As a result, three of the top 10 genes (20%) were mapped as the already known wing development and metamorphosis-related genes, indicating that the current approach of network analysis confers a high level of reliability and confidence in selecting genes. Wing development and metamorphosis-related genes with high betweenness centrality scores could be identified based on their importance in information flow in the altered gene-gene network during developmental stages.

**Table 4:** List of genes with wing development and metamorphosis-related genes and topological network parameters (Number of interaction partner and betweenness centrality).

Gene	Function	Number of partners	Betweenness centrality
arm	Wing	35	0.024
Cdc42	Wing	23	0.012
RasGAP1	Wing	18	0.006
unk	Wing	15	0.006
sqh	Wing	13	0.006
dom	Wing	12	0.520
par-1	Wing	7	0.001
osa	Wing	6	0.003
ksr	Wing	4	1.000
fu	Wing	3	0.001

### Comparative analysis of coleoptera genomes

**Fig 6:**
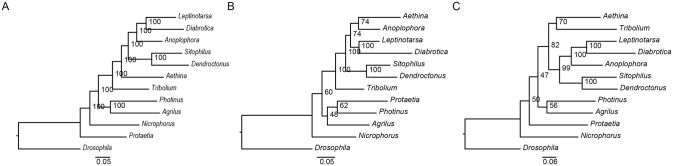
Genome comparison among representatives of coleoptera groups and coleoptera phylogeny. (**a**) 2,686,932 aa from 9,608 conserved orthologues, (**b**) 35,555 aa sites from 269 wing development orthologous, and (**c**)1,952 aa sites from 19 metamorphosis orthologous. The tree was reconstructed by IQ-Tree 1.5.1 with the best fit model (LG+F+G4). The *D. melanogaster* was used as outgroups, Branch lengths were optimized and node ages were estimated from 100 trees.

To explore the phylogenetic position of *P. b. seulensis* within coleoptera, we carried out phylogenetic analyses using the maximum likelihood method based on three aa sequence alignment sets of conserved orthologous genes, conserved wing development-related genes, and metamorphosis-related genes (Fig 6). Unaligned regions were removed using the Gblocks program (ver. 0.91b, used half gap option): 2,686,932 aa from 9,608 conserved orthologues, 35,555 aa sites from 269 wing development orthologous, and 1,952 aa sites from 19 metamorphosis orthologous. Each of the alignments contained 12 species as follows: *Aethina tumida*, *Agrilus planipennis*, *Anoplophora glabripennis*, *Asbolus verrucosus*, *Callosobruchus maculatus*, *Dendroctonus ponderosae*, *Diabrotica vigifera*, *Drosophila melanogaster*, *Ignelater luminosus*, *Leptinotarsa decemlineata*, *Nicrophorus vespilloides*, *Onthophagus taurus*, *Photinus pyralis*, *Protaetia brevitarsis*, *Sitophilus oryzae*, and *Tribolium castaneum*. The ML trees reconstructed using IQ-Tree 1.5.1 (Fig 6) show the different topology between three grouped genes. This implies that wing development-related genes and metamorphosis related-genes did not have the robust topology of the phylogeny. We also investigated which factors mostly influenced the topological difference between wing development and metamorphosis gene groups. The evolutionary forces imposed on the wing development gene group and metamorphosis gene group could influence the tree topology.

## Discussion

In this study, four stages of development (eggs, larvae, pupae, and adults) of *P. b. seulensis* were compared. A clearer understanding of the molecular mechanisms that regulate insect life cycles and development at all stages may help control this special functional gene by promoting wing development and deformation in a sustainable and environmentally-friendly approach. These results greatly increase the molecular resources available for research on other edible insects, and therefore providing a framework for understanding changes in gene expression levels during their development. All transcripts were constructed in four stages, obtaining 23,551 single genes with an average length of 300 bp. Approximately 23,494 sequences of the insects were annotated based on four major databases. Because about half of all single genes have not been previously annotated, the depth and analysis of Illumina sequencing in this study were improved compared to previous reports. Single genes are short in sequence or representative of very new genes; therefore, they do not significantly match the available databases. Seven of the top 10 annotated unigenes belong to the genus Coleoptera, emphasizing the preservation of these genes.

Although the full genome information of *P. b. seulensis* is insufficient, annotations of the four stages of development indicate that the insect has a high proportion of functional genes [49–52]. Edible insects exhibit typical morphological development characteristics, from eggs to five-stage larva to pupae and finally adulthood. Adults lay eggs and begin a new life cycle. During egg-to-larva transition, 2,189 up-regulatory genes and 6,152 down-regulatory genes were identified. Most of the top 10 genes, such as salivary cysteine-rich peptide precursor genes and skin protein genes RR-124–26 were related to immunization, digestion, and skin conversion. Additionally, this protein gene is ubiquitous in insects during their larval life. Insect cuticles are composed of cuticle proteins and chitin, which support and maintain the physical structure of organisms and act as natural barriers to external adverse conditions.

Most interestingly, we identified Cluster 4 as a significant gene group because it contains several wing formation and metamorphosis-related genes. General expression patterns of genes belonging to Cluster 4 were significantly down-regulated transiting from eggs to larva, whereas their expression level was reused as they went to pupae, therefore showing a pattern of expression-level maintenance even in adults. This phenomenon was also clearly revealed in the Kr-h1 gene, known to play an important role as a wing formation and metamorphosis-related gene. Additionally, most genes involved in 20E signaling and JH signaling showed a similar expression pattern as those in Cluster 4. However, these genes are known for their major function in wing formation and metamorphosis and it showed an up-regulation of gene expression in all stages of development. These expression patterns are distinct from the expression pattern of the genes belonging to Cluster 4. They act as factors that regulate and command signal conductors at a higher level of wing development and metamorphosis regulation.

Therefore, in this study, four developmental stages of transcriptome produced the genes and genome resources needed to investigate *P. b. seulensis*. Future analysis of genes related to insect development and death will help in control. Additionally, the richness of single genes and expression data obtained from this study provides information on the identification of genes involved in the development of *P. b. seulensis*.

## Conclusion

In this study, we explored gene expression abundance with respect to wing development and metamorphosis in *P. b. seulensis* based on the large-scale RNA-seq data obtained from every different developmental stage, namely eggs, larva, pupa, and adults. When conducting three-time repetitive RNA-seq data productions, the results consistently exhibited high reproducibility. The resultant *de novo* transcriptome assembly consists of 23,551 high-quality transcripts, which are approximately 96.7% covered; out of 8,545 expressed transcripts, 5,183 correspond to the possible orthologs of *Drosophila melanogaster*. Finally, we found 265 wing development genes, 19 metamorphosis-related genes, and 1,314 potential candidates. Out of the 1,598 genes, 1,594, except for four wing development genes, are included exclusively in Cluster 4 among 11 major clusters recognized on the basis of gene co-expression patterns along developmental stages. The function interaction network and the network centrality analyses showed that the 1,598 genes highlighted with the 284 wing development- and metamorphosis-related genes appear with high degree of betweenness centrality, indicating their key roles in molecular signaling pathways. The Cluster 4 genes are expressed most highly in eggs, moderately in pupae and adults, and lowest in larva, implying that they are closely related to the four development stages, eggs, pupa, adults, and larva. This study provides meaningful clues in elucidating the genetic modulation mechanism of wing development and metamorphosis in *P. b. seulensis*.

## Supporting information

S1 Table 23,551 unigenes and FPKM values in each developmental stages. (XLSX)

S2 Table Wing development related gene ontology and number of genes in *Protaetia brevitarsis seulensis*. (XLSX)

S3 Table Lists of wing development related genes. (XLSX)

S4 Table Lists of metamorphosis related genes. (XLSX)
